# Roles of gut microbiota and metabolites in overweight and obesity of children

**DOI:** 10.3389/fendo.2022.994930

**Published:** 2022-09-08

**Authors:** Shengan Zhang, Yanqi Dang

**Affiliations:** ^1^ Institute of Digestive Diseases, Longhua Hospital, China-Canada Center of Research for Digestive Diseases (ccCRDD), Shanghai University of Traditional Chinese Medicine, Shanghai, China; ^2^ School of Basic Medicine, Shanghai University of Traditional Chinese Medicine, Shanghai, China

**Keywords:** children, overweight, obesity, gut microbiota, metabolites

## Abstract

The prevalence of overweight and obesity in children and adolescents is an increasing public health problem. Pediatric overweight and obesity result from multiple factors, including genetic background, diet, and lifestyle. In addition, the gut microbiota and their metabolites play crucial roles in the progression of overweight and obesity of children. Therefore, we reviewed the roles of gut microbiota in overweight/obese children. The relationship between pediatric overweight/obesity and gut metabolites, such as short-chain fatty acids, medium-chain fatty acids, amino acids, amines, and bile acids, are also summarized. Targeting gut microbiota and metabolites might be a promising strategy for interventions aimed at reducing pediatric overweight/obesity.

## The epidemiological characteristics of pediatric overweight/obesity

Currently, the epidemic of pediatric overweight/obesity is one of the most concerning public health issues. In the past three decades, the prevalence of pediatric overweight/obesity has dramatically increased (1). Overweight/obesity in childhood increases the risk of diet-related noninfectious diseases, which are closely related to cardiovascular events in adulthood (2). Pediatric overweight/obesity is also associated with metabolic diseases (3) and depression (4).

In developed countries, the rates of pediatric overweight/obesity in boys and girls were 23.8% and 22.6% while the rates were 12.9% and 13.4% in developing countries in 2013, respectively (5). In UK, 25.5% children aged 10-11 were obese while 15.4% were overweight, but not obese and the rate of world-wide pediatric obesity is of concerning (6). Epidemiological investigation indicates that the prevalence of overweight/obesity among children and adolescents was 17.8% in the United States (7). It is also estimated that the rate of pediatric overweight/obesity has increased in China from 5.3% in 1995 to 20.5% in 2014 (8), which is positively related to socioeconomic conditions (8, 9). In 2019, 11.1% and 3.6% of Chinese children under six years of age were overweight and obese, respectively, and 34.3% and 7.9% of Chinese children aged 6-17 years were overweight and obese, respectively (10), indicating that one-quarter of Chinese children are overweight/obese (11). In addition, there are significant regional and sex differences in the incidence of pediatric overweight/obesity, with the rate in boys generally higher than in girls. For eastern, southern, northern, central, and western China, Children from southern and northern had the lowest and highest prevalence of overweight and obesity, respectively (12).

Studies have demonstrated a strong correlation between childhood and adult obesity. Compared to non-obese children, obese children are five times more likely to be obese in adulthood (13). Therefore, intervention and research on pediatric overweight/obesity are of great importance.

## The relationship between diets and obesity

Excessive energy intake and conversion to lipid accumulation in the body are the main causes of obesity. Therefore, a sensible diet is a key to avoiding obesity. Dietary factors, including high fat, high fructose, and other unhealthy dietary patterns (14, 15), are important obesity-causing factors in addition to genetic background, sleep, mental state, and exercise habits (16). A study found that from childhood to adolescence, the dietary quality decreases significantly in South Carolina, especially the declines in fruit, vegetable and total dairy intake followed by increasing protein intake (17). Another study also found that weight gain is positively related to the intake of potato chips, sugary beverages and red meats, and is inversely related to the intake of vegetables, fruits, nuts, and yogurt (18). In the past thirty years, the transition from a traditional low fat, high carbohydrate diet to a high fat and low carbohydrate diet has been associated with a dramatic increase in the risk of obesity, type 2 diabetes, cardiovascular diseases and colon cancer in China (19). Furthermore, Obesity is also positively correlated to fat intake, especially a diet rich in long-chain saturated fatty acids, which promotes inflammation of multiple organs (20). Children’s sugar intake, such as fructose, is of concern. Dietary fructose promotes hepatic *de novo* lipogenesis (DNL) *via* acetate from the gut microbiota (21–23). The World Health Organization recommends that the average daily sugar intake should not exceed 25 g (24, 25). Despite the decrease in the consumption of sugary beverages in children, it is still a critical source of energy intake (26) and is positively related to obesity (27). Although the restriction of carbohydrate intake may benefit short-term body weight control and improve the blood glucose levels of overweight/obese patients, long-term carbohydrate restriction may lead to a reduction in dietary fiber intake and aggravate fatigue, making it difficult to maintain (28). Therefore, further studies on the safety and effectiveness of carbohydrate restriction in children and adolescents are warranted.

## The relationship between pediatric overweight/obesity and gut microbiota

Gut microbiota is regarded as an essential factor regulating the process of overweight/obesity as it participates in the energy metabolism of the host and maintains homeostasis of the internal environment. *Firmicutes*, *Bacteroidetes*, *Proteobacteria*, and *Actinobacteria* are the most dominant bacterial phyla in the human gut microbiota (29, 30). Moreover, low-abundance *Verrucomicrobia* shows potential benefits on metabolism. With the development of high-throughput sequencing, many studies have shown that an imbalance in gut microbiota is closely related to the progression of overweight/obesity in children (31) (**Table 1**). Both simple and genetic obesity (such as Prader-Willi Syndrome, PWS) can be relieved by adjusting the dietary structure, partly targeting the gut microbiota (14). Improving the imbalance of the gut microbiota may be an effective way to intervene against overweight/obesity in children (48).

**Table 1:** Relationships between gut microbiota and pediatric overweight/obesity.

Phyla	Species/families		Pediatric overweight/obesity	Reference
*Firmicutes*			↑	32
*Firmicutes*	*Lactobacillus*		↑	33
*Firmicutes*	*Clostridium*		↑	34
*Bacteroidetes*			↓	35, 36
*Bacteroidetes*	*Bacteroides fragilis*		↑	37
*Verrucomicrobia*			↓	38, 39
*Verrucomicrobia*	*Akkermansia muciniphila*		↓	40
*Proteobacteria*			↑	41, 42
*Proteobacteria*	*Enterobacteriaceae*		↑	43
*Proteobacteria*	*Escherichia coli*		↑	44
*Actinobacteria*			↓	45, 46
*Actinobacteria*	*Bifidobacterium*		↓	47

Symbol ↑ indicates that the gut microbiota of this phylum or species have a higher abundance in pediatric overweight/obesity and symbol ↓ indicates that the gut microbiota of this phylum or species have a lower abundance in pediatric overweight/obesity.

### Firmicutes, bacteroidetes, and firmicutes: Bacteroidetes ratio

*Firmicutes* and *Bacteroidetes* are the two most abundant phyla in human gut microbiota. *Firmicutes* are gram-positive bacteria with low GC content, including *Clostridium*, *Lactobacillus*, and *Coprococcus*. *Bacteroidetes* mainly contain *Bacteroides*, *Prevotella*, and *Desulfuribacillus*. It is generally believed that the abundance of *Firmicutes* in overweight/obese adults increases, whereas the abundance of *Bacteroidetes* decreases (48, 49), resulting in an increase in *Firmicutes: Bacteroidetes* ratio. The same result was observed in the gut microbiota of overweight/obese children (32). Compared to normal-weight children, the abundance of *Firmicutes* in overweight/obese children is positively correlated with body mass index (BMI) (47, 50) whereas the abundance of *Bacteroidetes* is negatively correlated with BMI (35, 36). Therefore, *Firmicutes: Bacteroidetes* ratio is positively correlated with overweight/obesity in children (31, 51).

Although *Firmicutes: Bacteroidetes* ratio is a common index to measure the structure of the gut microbiota, heterogeneity still exists between this ratio and overweight/obesity. Indiani et al. found that the abundance of *Bacteroidetes* was increased in overweight/obese children along with a decrease of *Firmicutes* only in some cohorts (35). Another systematic review also indicated that the *Firmicutes: Bacteroidetes* ratio was not related to pediatric overweight/obesity (52), which might be the result of potential heterogeneity in the roles of *Firmicutes* and *Bacteroidetes*. Members of *Firmicutes* show a greater variation in abundance than *Bacteroidetes* (31). Since the structure of the gut microbiota in children is still under development, the composition is unstable. Consequently, changes in gut microbiota in overweight/obese children can show an individualized trend (53). Compared to differences in gut microbiota abundance at the phylum level, differences at the genus level and specific metabolites may be more commonly and directly associated with pediatric overweight/obesity (31).

Further research has shown that the abundance of *Firmicutes* was closely related to inflammatory levels and positively correlated to serum tumor necrosis factor α (TNF-α) levels in obese children (50). The epigenetic effects of *Firmicutes* are also concerning. During pregnancy, a high *Firmicutes* abundance is associated with DNA methylation of genes related to lipid metabolism, inflammatory response, and obesity (54). *Lactobacillus*, a key genus of *Firmicutes*, is usually considered a probiotic with a long history of application (55). However, the link between *Lactobacillus* and pediatric overweight/obesity remains paradoxical. The abundance of *Lactobacillus* is positively related to the risk of pediatric overweight/obesity (33), and fecal *Lactobacillus* concentrations in children are associated with serum C-reactive protein (51). *Lactobacillus* colonization predicts a higher risk of overweight/obesity in infants and children (56). Certain members of *Lactobacillus*, such as *Lactobacillus paracasei* are protective factors against obesity in children with an unhealthy diet (57, 58). *Clostridium* is positively associated with BMI in children (34), and is more significant in young adults (59). *Bacteroides fragilis* is significantly associated with a higher BMI z-score in children, contributing to weight gain during childhood (37).

### *Verrucomicrobia* and *Akkermansia muciniphila*

The abundance of *Verrucomicrobia* is relatively low in the human gut microbiota. However, recent research has shown that the abundance of *Verrucomicrobia* was low in obese children (38, 39, 60), and it has vital benefits. Hence, it is a potential probiotic against metabolic inflammation and obesity (61). The anaerobic bacterium *Akkermansia muciniphila* is the only known member of *Verrucomicrobia* in human’s intestinal tract (62). Overweight/obese children have lower *Akkermansia muciniphila* abundance (40), which is also observed in obese adults (63).

*Akkermansia muciniphila* is a pivotal species in the intestinal mucous layer that produces acetate and propionate (62). The decreased abundance of *Akkermansia muciniphila* may lead to high intestinal permeability (60), and the protection of interleukin-36 against obesity is partly realized by promoting *Akkermansia muciniphila* levels (64). *Akkermansia muciniphila* can prevent diet-induced obesity and demonstrate hepatoprotective effects by downregulating the metabolism of tyrosine, phenylalanine, tryptophan, and their intermediates, all of which have adverse effects. In addition, *Akkermansia muciniphila* weakens acetyl-CoA oxidation in the citric acid cycle and promotes ketogenesis (63). Surprisingly, oral vancomycin administration can lead to an increase in *Akkermansia muciniphila* (65). Despite the relatively low abundance of *Verrucomicrobia* and *Akkermansia muciniphila*, multiple studies have indicated that their abundance is strongly associated with pediatric and adult obesity.

### Proteobacteria

*Proteobacteria* include purple photosynthetic bacteria and their relatives. There is a high abundance of *Proteobacteria* in the feces of obese children, and these bacteria have a significant positive correlation with BMI levels (41, 42). *Proteobacteria* are also relatively abundant among malnourished children and contain many potentially pathogenic species that induce immature intestines or potentially high inflammatory burdens (66). Of note, physical exercise significantly reduces the abundance of *Proteobacteria* in obese children (67).

*Proteobacteria* mainly include *Alphaproteobacteria*, *Betaproteobacteria*, *Gammaproteobacteria*, *delta/epsilon* subdivisions, and *Zetaproteobacteria*, among which *Gammaproteobacteria* participates in the metabolism of choline, and it has a high abundance in overweight/obese children with non-alcoholic fatty liver disease (NAFLD) (68, 69). *Enterobacteriaceae*, a member of *Gammaproteobacteria*, is commonly observed in overweight adolescents (43). The abundance of *Escherichia coli* is also significantly higher in obese children (44). *Escherichia coli* can produce alcohol as an endotoxin that can impair hepatic metabolism (60). Endogenous alcohol exaggerates the metabolic burden on the liver, which is of great importance in obese children with non-alcoholic hepatitis (NASH) (42). However, it has been shown that a decrease in *Escherichia coli* is related to higher lipopolysaccharide (LPS) levels (59).

### *Actinobacteria* and *Bifidobacterium*

The abundance of *Actinobacteria* is negatively correlated with children’s BMI (45, 46). *Actinomycetales*, an order of *Actinobacteria*, is positively related to hemoglobin concentration in anemic infants (70). *Bifidobacterium* is a well-known probiotic of *Actinobacteria*, which can promote the development and maturation of infant intestinal mucosa, thereby lowering the incidence of diarrhea (71). Additionally, *Bifidobacterium* inhibits the growth of adverse microbiota *via* competitive colonization, demonstrating an antagonistic relationship with *Enterobacteria* and *Enterococci* (72). The abundance of *Bifidobacterium* is also high in the intestines of vegetarians (45). *Bifidobacterium* carries genes encoding bacterial bile salt hydrolase (BSH) (73), which increases the excretion of bile acid and simultaneously inhibits the absorption of cholesterol. Furthermore, *Bifidobacterium* also produces short-chain fatty acids (SCFAs) (45).

The abundance of *Bifidobacterium* is negatively correlated with BMI in children. Studies have shown that the abundance of *Bifidobacterium* in overweight/obese children is significantly lower than that in children of normal weight, and it is hypothesized to participate in fat accumulation and obesity (47). During weight loss, *Bifidobacterium* abundance rebounds (58). *Bifidobacterium infantis* metabolizes human milk oligosaccharides (HMO) and suppresses HMO uptake by pathogenic microbes. Increased levels of SCFAs stimulate the immune response and regulate the function of pancreatic β cells (74). Introducing *Bifidobacterium*, as a dietary supplement, is one strategy that reduces pediatric obesity (75). Treatment with *Bifidobacterium breve BR03* and *B632* significantly improves insulin sensitivity in obese children and adolescents (76). Supplementation with *Bifidobacterium pseudocatenulatum CECT 7765* can improve the inflammatory response in obese children with insulin resistance (IR) (77). Interestingly, other probiotics, such as *Lactobacillus casei*, can also upregulate the abundance of *Bifidobacterium* in obese children, exhibiting a synergistic effect (78).

## The relationship between pediatric overweight/obesity and gut metabolites

**Figure 1:**
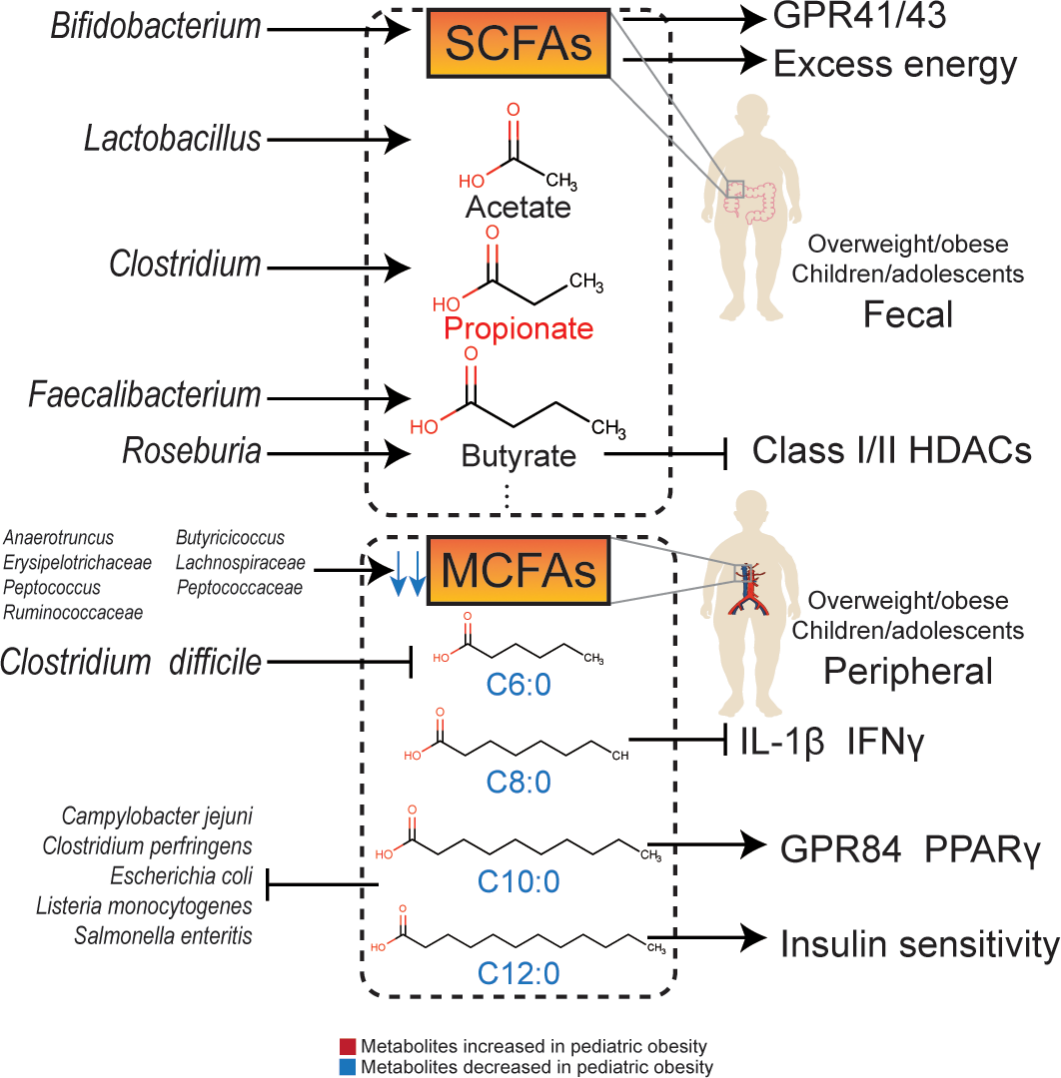
The relationship and molecular signaling between gut microbiota and their metabolites with obesity.

**Figure 2:**
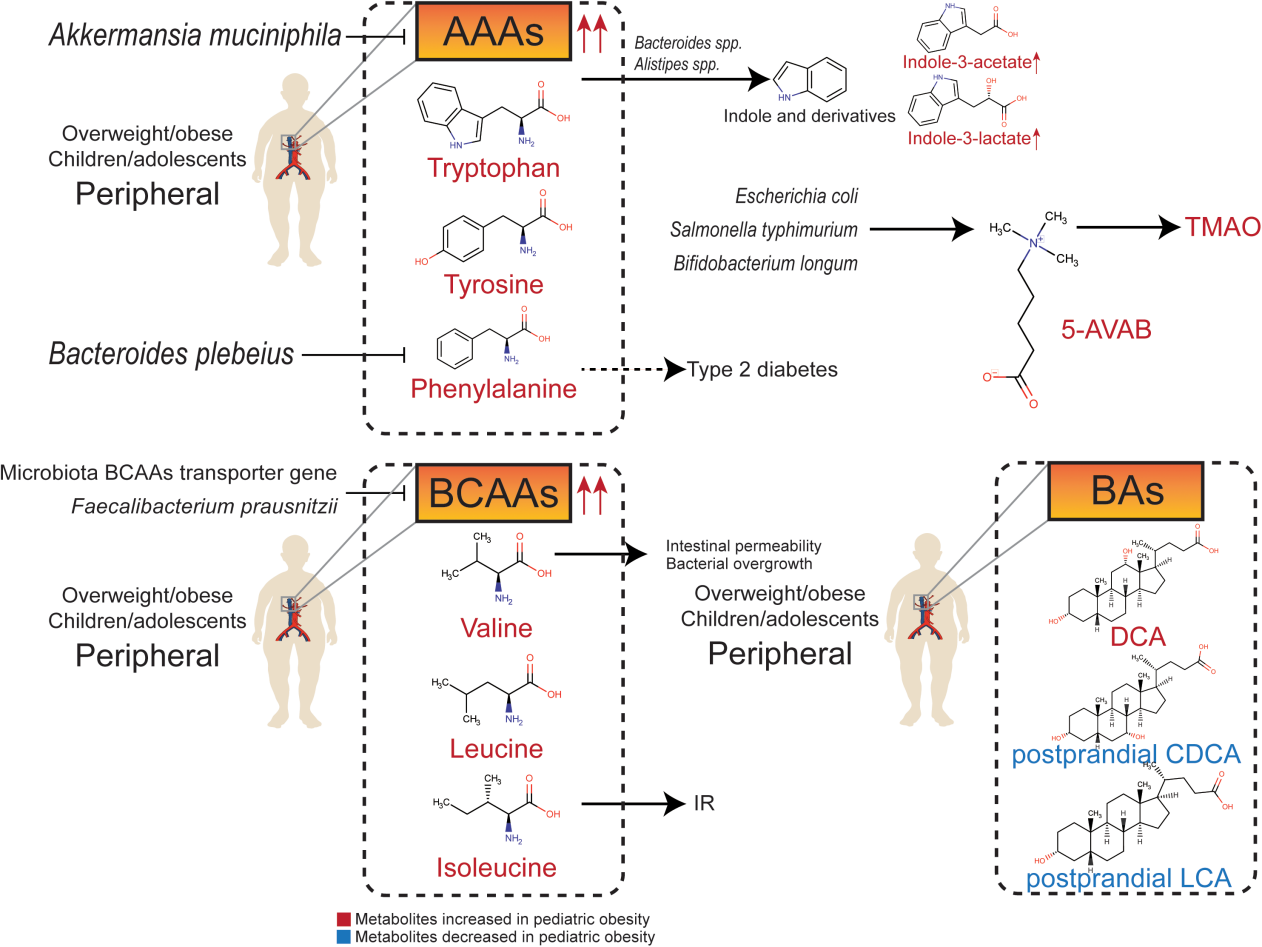
Relationship between gut metabolites (amino acids, amines, and bile acids) and pediatric overweight/obesity.

The effects of the gut microbiota are mainly mediated by the absorption and distribution of their metabolites (79). The gut microbiota can produce dozens of metabolites that enter the bloodstream to have a systemic effect on the host (80). There are new evidences to support the association between obesity and these metabolites, including SCFAs, medium-chain fatty acids (MCFAs) (**Figure 1**), amino acids, amines, and bile acids (**Figure 2**). The gut microbiota and metabolites in obese people can significantly change compared to those in people with normal weight. A decrease in the abundance of *Bacteroides thetaiotaomicron*, which is capable of metabolizing glutamate, results in a higher risk of obesity, and the gut microbiota in obese adolescents has a stronger ability to oxidize carbohydrates (81). Therefore, it is possible to intervene in overweight/obesity by targeting the gut microbiota and its metabolites (82). Changes in the gut microbiota have been shown to be related to pediatric obesity and NAFLD. Biosynthesis of SCFAs, amino acids, and LPS is inversely correlated with IR, whereas peptidoglycan biosynthesis pathways are positively correlated with IR (83).

### Short-chain fatty acids

SCFAs are endpoint products of indigestible complex carbohydrates, such as dietary fiber and resistant starch, fermented by intestinal flora (84). These mainly include organic acids whose carbon chains have less than six carbon atoms, such as acetate, propionate, butyrate, isobutyrate, valerate and isovalerate. Among them, acetate, propionate, and butyrate have the highest concentrations, with an approximate proportion of 3:1:1. SCFAs can regulate the function of adipose tissue and be used as substrates for gluconeogenesis and DNL in the liver (85). Plasma acetate, propionate, and butyrate levels are associated with BMI and visceral fat in adolescents (81). Increased levels of SCFAs may prevent gastrointestinal dysfunction, obesity, and type 2 diabetes (86).

Acetate can combine with G-protein-coupled receptor 41 (GPR41) and G-protein-coupled receptor 43 (GPR43) to regulate metabolism (87). In addition, acetate can be converted into acetyl-CoA, which enters the tricarboxylic acid cycle and participates in energy metabolism. Acetate may also affect metabolism by regulating AMP-activated protein kinase (AMPK) phosphorylation, and increase fatty acid synthesis through epigenetic mechanisms. Serum acetate levels are negatively correlated with fasting insulin levels (88). The concentration of acetate in the feces of obese children is low and is normalized after dietary intervention (14) or treatment with *Lactobacillus casei*, an acetate-producing bacterium (78). In contrast, the concentration of propionate in feces is positively correlated with the waistline of female adolescents, indicating an adverse metabolic effect (89). In obese adolescents, the rate of acetate in peripheral circulation is lower than that in lean individuals (90). However, after intervention with *Bifidobacterium breve BR03* and *B632*, the concentration of fecal acetate in obese children was lower than that in a placebo group. Since *Bifidobacterium breve* is an acetate-producing strain, the possibility of acetate being absorbed and utilized by other acetate-dependent species needs to be considered (76).

Propionate is a metabolite partly from *Clostridium*, which is positively related to pediatric overweight/obesity (91). The concentration of propionate in feces is positively related to fasting blood glucose and glycosylated hemoglobin levels (92), and also related to an increased risk of type 2 diabetes (93). Compared to breastfed children, children who underwent no exposure to human milk have higher concentrations of propionate in feces (94). The serum propionate level is positively associated with BMI in obese children (81), and the fecal propionate concentration is also significantly increased in overweight/obese children (95, 96), which is consistent with that in overweight/obese adult (97). Moreover, concentrations of lactate, as an substrate of propionate metabolism, (98) are decreased in obese children, indicating that propionate metabolism was activated by gut microbiota (99).

Butyrate is an effective regulator of energy metabolism and immune functions (100). *Faecalibacterium prausnitzii* and *Roseburia hominis* are important butyrate-producing strains (101). The butyrate-producing ability of *Faecalibacterium prausnitzii* is essential for gastrointestinal and metabolic health (98). A decrease in butyrate-producing strains in obese children has been observed (83), and fecal butyrate concentration is negatively related to gut microbiota diversity, which also affects intestinal permeability (102). In addition to GPR41/43, butyrate can regulate metabolism through β-oxidation (103) and inhibit class I/II histone deacetylases (HDACs) (104). Butyrate can also promote the secretion of glucagon-like peptide-1 (GLP-1), thereby enhancing insulin sensitivity (105).

The concentration of SCFAs in obese children is low (106), however, some studies have shown high levels of acetate, propionate, butyrate, and isovalerate in obese children (31, 38, 99), which are positively correlated with BMI z-scores (31). Despite the potential benefits of SCFAs, the overall function of SCFAs in pediatric overweight/obesity remains unclear. SCFAs can be double-edged swords: on the one hand, excess SCFAs are an extra energy source, participating in the process of pediatric overweight/obesity; while, on the other hand, SCFAs promote insulin excretion *via* the GPR41/43 pathway (107).

### Medium-chain fatty acids

The relationship between pediatric overweight/obesity and MCFAs were verified by epidemiological evidences. MCFAs seem to be a protective factor for pediatric overweight/obesity. A study has shown that the concentration of caproic acid (C6:0) in serum, which is also known as hexanoic acid, was significantly decreased in obese children (108). Caproic acid is also significantly decreased in patients suffering from *Clostridium difficile* infection, which is positively related to pediatric overweight/obesity (109). The capric acid (C10:0) level in obese children is significantly lower than that in normal-weight children (110). Compared to neonates with low adiposity, the dodecanoic acid (C12:0) level in neonates with high adiposity is significantly lower (100).

It is difficult for MCFAs from food to reach the cecum completely due to the presence of gut microbiota. Recently, an animal experiment conducted by Gregor et al. (111) observed MCFAs production by gut microbiota. MCFAs not only metabolize quickly to produce energy in the colon but can also enter the liver or adipose tissue. Gut microbiota, including *Erysipelotrichaceae*, *Peptococcaceae*, *Anaerotruncus*, *Butyricicoccus*, *Lachnospiraceae*, *Peptococcus*, and *Ruminococcaceae* are positively correlated with the concentrations of MCFAs in the cecum. In addition, studies have shown that *Caproiciproducens*, *Pseudoramibacter*, *norank_f_Eubacteriaceae*, and *Oscillibacter* catabolize lactate into MCFAs (112).

Zhao et al. found that caprylic acid (C8:0) downregulated the serum levels of interleukin-1β (IL-1β) and interferon γ (IFNγ) and increased the abundance of *Lachnoclostridium*, *Roseburia*, and *Prevotella_9*, activating GPR43 and improving intestinal permeability (113). Capric acid (C10:0) can upregulate GPR84 and peroxisome proliferator-activated receptor γ (PPARγ) (114). Dodecanoic acid (C12:0), also known as lauric acid, can improve insulin sensitivity (100). Although the difference between SCFAs and MCFAs is only a few carbon atoms, they demonstrate different immune regulatory mechanisms. SCFAs downregulate levels of IL-1β, IL-6, and TNFα *via* toll-like receptor 4 (TLR4), whereas MCFAs may enhance the inflammatory response through TLR2 (114).

Apart from immune regulation, MCFAs also inhibit various intestinal pathogenic microorganisms. Both capric acid and dodecanoic acid inhibit the growth of *Listeria monocytogenes*, *Clostridium perfringens*, *Escherichia coli*, *Salmonella enteritis*, and *Campylobacter jejuni* (115). Dodecanoic acid can also inhibit the growth of *Staphylococcus aureus* (116).

MCFAs have potential therapeutic effects on genetic obesity during the childhood. Dietary supplementation with MCFAs can alleviate adipose accumulation in mice (117). More importantly, patients with PWS diagnosed in early infancy responded with the improvement of motor development and nutritional conditions after dietary supplementation with MCFAs (118).

### Amino acids and amines

In addition to SCFAs and MCFAs, metabolites of the gut microbiota include nitrogenous compounds produced from amino acids. There are two main pathways of amino acid metabolism: deamination to carboxylic acid and ammonia, and decarboxylation to CO_2_ and amine. The most abundant end products of catabolism are SCFAs (119). Other metabolites, such as amino acids and amines, also affect host health.

Methionine and cysteine are sulfur-containing amino acids that are catabolized into H_2_S and methyl mercaptan by *Bacillus* and *Bifidobacterium* (119). Cysteine exhibits anti-diabetic properties. *Parasutterella* sp. is one of the main consumers of cysteine. The abundance of *Parasutterella* sp. is negatively related to the serum concentration of cysteine, and is significantly downregulated in weight-loss-obesity patients (120). Methionine biosynthesis mediated by gut microbiota is associated with atherosclerosis in obese children (121). The ability to utilize sulfur-containing compounds is essential for gut microbiota. Sulfatases and radical S-adenosyl-L-methionine synthetase play vital roles in the colonization of microorganisms in the intestinal tract (122).

The ability of intestinal flora to metabolize aromatic amino acids (AAAs) and branched-chain amino acids (BCAAs) is significantly increased in obese individuals (123). AAAs, BCAAs, and their downstream metabolites produced by the gut microbiota can interfere with glucose homeostasis and contribute to IR (124).

The AAAs mainly include tryptophan, phenylalanine, and tyrosine. They are essential to the intestinal tract and greatly contribute to gut dysfunction. The abundance of tryptophan derivative metabolites in plasma is significantly altered in obese children (125). Indole is produced by gut microbiota-mediated fermentation of tryptophan, which is an obesity-promoting metabolite that targets the brain-gut axis (126). Harmful tryptophan metabolites, such as indole derivatives, are produced partly by the tryptophanase of *Bacteroides* spp. and *Alistipes* spp. (14). The serum concentrations of indole-3-lactate and indole-3-acetate in obese children are increased (127). Furthermore, obese children have higher levels of serum phenylalanine, which is positive associations with the risk of type 2 diabetes (128), whereas, *Bacteroides plebeius* is negatively related to serum phenylalanine concentration in children (129). Both tryptophan metabolism and phenylalanine metabolism regulated by gut microbiota are significantly downregulated after dietary intervention in both simple and genetic obese children (14). The serum concentration of tyrosine is significantly decreased in obese children with substantial weight reduction (130). Furthermore, the potential probiotic *Akkermansia muciniphila* can decrease serum levels of diverse intermediates in tryptophan, phenylalanine, and tyrosine metabolism in obese individuals (63).

BCAAs include leucine, isoleucine, and valine. The proportion of isoleucine in total protein intake is positively correlated with BMI (21), so dietary restriction of BCAAs may help prevent childhood obesity and IR (131). Plasma BCAAs levels are significantly higher in obese children and adolescents, and are consistent in children from multiple ethnic backgrounds (132–136), which can predict the risk of IR and metabolic syndrome independently (133, 134). Metabolic analysis based on BCAAs can predict hepatic steatosis grading in high-risk children and adolescents (137). Valine metabolites are associated with increased intestinal permeability and bacterial overgrowth in the small intestine in children (138). The transporter gene of BCAAs is negatively correlated with serum levels of BCAAs in adolescents, suggesting that the gut microbiota may reduce the level of BCAAs in the peripheral circulation. In contrast, *Faecalibacterium prausnitzii* regulates IR through BCAAs metabolism (139).

Trimethylamine N-oxide (TMAO) is a hazardous metabolite that originates from trimethylamine, which is produced in the liver. The urine concentration of TMAO is significantly higher in children with simple and genetic obesity (14). 5-amino valeric acid betaine (5-AVAB), also known as δ-Valerobetaine, is a precursor of TMAO produced by *Escherichia coli*, *Salmonella typhimurium*, and *Bifidobacterium longum* (140). 5-AVAB affects the carnitine shuttle system of mitochondria, lowering the ability of hepatocytes to oxidize fatty acids, and resulting in lipid accumulation. Obese individuals have higher plasma 5-AVAB concentrations, which are also positively related to hepatic steatosis in children with NAFLD (141).

### Bile acids

Cholesterol is oxidized to primary bile acid in hepatocytes through classical and alternative pathways to produce cholic acid (CA) and chenodeoxycholic acid (CDCA), respectively. CA and CDCA combine with glycine and taurine to generate glycocholic acid (GCA), taurocholic acid (TCA), glycochenodeoxycholic acid (GCDCA), and taurochenodeoxycholic acid (TCDCA), respectively. Primary bile acids enter the intestinal tract *via* the biliary system. Glycine and taurine are dissociated under the catalysis of gut microbiota BSH, and a series of secondary bile acids are formed, such as lithocholic acid (LCA), hyocholic acid (HCA), hyodeoxycholic acid (HDCA), deoxycholic acid (DCA), and ursodeoxycholic acid (UDCA). Bile acids are released into the small intestine to promote the intake of lipids and fat-soluble vitamins. The imbalance in the composition and spectrum of bile acids is related to obesity. Therefore, the regulation of bile acids may be a potential strategy to intervene against obesity (142).

Most of the secondary bile acids produced by intestinal microflora can enter the liver through the portal vein, and only a few of them are excreted through feces. Therefore, secondary bile acids can regulate hepatic steatosis and NAFLD, which is highly associated with obesity. A sex-specific study in mice exposed to high fructose showed that both primary and secondary bile acids decreased in female mice, but there was no similar trend in male mice (143). In overweight children with NAFLD, serum total bile acids, especially glycine-binding bile acids such as GCDCA, GCA, glycodeoxycholic acid (GDCA), and glycoursodeoxycholic acid (GUDCA), are lower than those in cohorts with normal BMI (144). Compared to obese children without NAFLD, obese children with NAFLD have higher levels of serum total bile acids and glycine-bound bile acids (145).

Secondary bile acids have complex and unelucidated biological functions. Studies have illustrated that secondary bile acids regulated by high fat diet has adverse metabolic effects (146). DCA tends to increase in diet-related or genetic childhood obesity (147). Contrary to previous study in obese adults (148), levels of postprandial bile acids are significantly lower in obese adolescents. Serum levels of non-12-OH bile acids, including CDCA and LCA, and intermediates in bile acids synthesis are lower in adolescents with obesity (149). UDCA is a drug used for the treatment of NAFLD, while low DCA is considered to be characteristic of a healthy metabolic spectrum of bile acids (73). UDCA supplementation can control diet-induced obesity in prenatally malnourished mice (150). In obese mice, the abundance of *Lactobacillaceae* and *Lachnospiraceae* producing secondary bile acids is higher and leads to higher levels of LCA and DCA (151). The proportion of non-12-OH bile acids, including HCA, HDCA, glycohyodeoxycholic acid (GHDCA), UDCA, GUDCA, and CDCA, in total bile acid, is significantly lower in people with high BMI, indicating that non-12-OH bile acids may contribute to the process of obesity. In addition, the ratio of CA and DCA is significantly higher, and the ratio of CDCA and UDCA is significantly lower in the population with high BMI, while fecal total bile acid is not significantly different in those with high BMI group compared with healthy people (152).

## The roles of gut microbiota in early life

The gut microbiota of obese patients differs from that in healthy people. Abnormalities in the gut microbiota and their metabolites are involved in the occurrence of obesity. An early difference in fecal microbiota in children may predict the occurrence of overweight, as this has a profound impact on the function of the digestive system (153). However, a single acquired factor or genetic factor is not sufficient to fully explain the changes in gut microbiota and metabolites in overweight/obese children. The adaptive changes in the structure of gut microbiota in early life are also related to long-term health problems, such as mucosal immune development and height retardation (154). Factors such as mode of delivery, diet, and breastfeeding can cause changes in the gut microbiota, which in turn can increase the risk of pediatric overweight/obesity (155). Therefore, gut microbiota in the early stages of life should play an important role in overweight and obese children.

Maternal gut microbiota has a long-lasting effect on the gut microbiota and metabolites of offspring. One of the key determinants is that *Bacteroides* abundance is reduced and delays colonization (156), which results in lower microbial genes associated with amino acids and nucleic acids metabolism, and higher genes associated with fatty acid metabolism and amino acid degradation (157). In addition, despite the low abundance of fungi in the gut microbiota, fungal host phenotypes can be transferred from parents to offspring, and fungal diversity and species composition in offspring may develop towards the fungal community of parents (158).

Breastfeeding is independently associated with infant gut microbiota diversity, which benefits the infant’s immune system by specifically providing nutrients to the microbes to form healthier immune-microbe relationships (159). For instance, deficiency of *Bifidobacterium* and its HMO-utilizing gene is related to systemic inflammation and immune dysregulation in early life. Feces from *Bifidobacterium infantis EVC001*-supplemented infants are rich in indole lactate and indole-3-lactate, which upregulate Galectin1 expression during Th2 and Th17 cell polarization (160). Supplementation with *Lactobacillus rhamnosus HN001* in mothers during pregnancy or breastfeeding can reduce eczema and allergy rates in babies (161). Maternal obesity may also affect the ability of offspring gut microbiota to metabolize BCAAs (135).

## Summary and outlook

While the structural characteristics of gut microbiota in obese children have been described in detail, there is still variation in the abundance of some bacteria. Changes in metabolic function caused by these variations need to be considered. Although probiotics are generally believed to be beneficial to the metabolism of children, current studies have not reached a consensus. An Iranian study found that probiotics could improve liver function in children with obesity. However, other studies have found that probiotics seem to increase obesity in Hispanic adolescents (162). Therefore, it is necessary to screen for probiotics in the treatment of childhood obesity.

Numerous mechanisms that associate intestinal flora with the risk of obesity and metabolic disorders are based on the findings of rodent models, but the structure of intestinal flora in rodents is quite different from that in humans (163). On the other hand, at present, research on gut microbiota and metabolites is still mainly focused on obese adults, while the research on obese children is relatively deficient. Due to the large differences in the structure of the gut microbiota between adults and children, future cohort studies on gut microbiota and metabolites in obese children are required.

## Author contributions

YD conceived, designed, and supervised the manuscript. SZ wrote the paper. YD edited and revised the paper. All authors reviewed and approved the final manuscript. All authors contributed to the article and approved the submitted version.

## Funding

This work was supported by the Shanghai Rising-Star Program (21QA1409000), and the Shanghai Frontier Research Base of Disease and Syndrome Biology of Inflammatory cancer transformation (2021KJ03-12).

## Conflict of interest

The authors declare that the research was conducted in the absence of any commercial or financial relationships that could be construed as a potential conflict of interest.

## Publisher’s note

All claims expressed in this article are solely those of the authors and do not necessarily represent those of their affiliated organizations, or those of the publisher, the editors and the reviewers. Any product that may be evaluated in this article, or claim that may be made by its manufacturer, is not guaranteed or endorsed by the publisher.
